# Mini-open lateral retropleural thoracic discectomy approach

**DOI:** 10.3171/2022.3.FOCVID2217

**Published:** 2022-07-01

**Authors:** Juan S. Uribe, Gennadiy A. Katsevman, Clinton D. Morgan, Gabriella M. Paisan, Laura A. Snyder

**Affiliations:** Department of Neurosurgery, St. Joseph’s Hospital and Medical Center, Barrow Neurological Institute, Phoenix, Arizona

**Keywords:** lateral retropleural approach, thoracic disc herniation, mini-open approach

## Abstract

The lateral retropleural approach provides an eloquent, mini-open, safe corridor to address various pathologies in the thoracolumbar spine, including herniated thoracic discs. Traditional approaches (e.g., transpedicular, costotransversectomy, or transthoracic) have their own benefits and pitfalls but are generally associated with significant morbidity and often require instrumentation. In this video, the authors highlight the retropleural approach and its nuances, including patient positioning, surgical planning, relevant anatomy, surgical technique, and postoperative care.

The video can be found here: https://stream.cadmore.media/r10.3171/2022.3.FOCVID2217

## Transcript

This video will demonstrate the mini-open lateral retropleural thoracic discectomy approach.

### 0:26 Patient History and Imaging.

The following patient presented with thoracic myelopathy and was found to have a large thoracic disc herniation. Given the size of the disc herniation and patient’s symptoms, he was taken to surgery for thoracic discectomy.

### 0:38 Patient Positioning and Incision Planning.

The patient was placed in a lateral decubitus position with the surgeon standing behind the patient. We utilize neuromonitoring with motor evoked potentials (MEPs) and somatosensory evoked potentials (SSEPs) for every case. A needle is placed in the spinous process at the level of the disc space of interest, as noted in the inset in the top left corner.^1^ The needle is placed under fluoroscopy by the spine surgeon at the time of surgery. This provides added orientation throughout the case regarding the target disc space. The disc space of interest—as well as the vertebral body above and the vertebral body below—are all marked out under fluoroscopy. A 6-cm incision is offset posteriorly and ideally centered over a rib that will be later harvested for autograft.^2^ It is important to note the various layers that will be encountered throughout the superficial dissection until reaching the rib, including the skin, fat, latissimus dorsi, external oblique, and intercostal muscles.^3^

### 1:35 Incision and Rib Dissection.

Incision is made sharply with a scalpel and continued with Bovie electrocautery until the rib is exposed. Various different instruments are available to strip the periosteum over the rib and to achieve circumferential dissection. Care is taken to avoid pleural violation underneath the rib during the dissection process, and to preserve the neurovascular bundle at the inferior portion of the rib. It is important to carry the rib dissection further than the incision, and an assistant can be useful in retracting the skin and soft tissue to increase the amount of anterior-posterior exposure. Using a combination of Penfield no. 1, rib dissectors, Cobb elevators, and curved curettes, the rib is circumferentially dissected. A rib cutter is utilized for rib resection, and a rongeur can be used to extend amount of resection if needed. The posterior remnant of the rib will limit perpendicular retractor placement at the disc space, so it is important to be aggressive with the posterior aspect of the rib. Once circumferential release of about 6 cm of rib is achieved, the rib is resected en bloc and preserved to be later used as morselized autograft.

### 2:42 Retropleural Dissection.

Immediately after rib resection, the parietal pleura and endothoracic fascia are visualized. Using a combination of sharp and blunt dissection, the layer between the parietal pleura and endothoracic fascia is developed until reaching the rib head.^4^ Again, care is taken not to violate the pleura. In the event of a pleural violation, a piece of Gelfoam can be placed over the defect to prevent protrusion of the lung through the pleural lining.

### 3:08 Retractor Placement and Rib Head Removal.

Once there is sufficient dissection, a retractor is placed into the field with the handles away from the surgeon. Additional fluoroscopy images are taken to center the retractor over the disc space, including the pedicle below and the anterior portion of the central canal. Bovie electrocautery is used to isolate the rib head, and an ultrasonic bone aspirator is used for rib head removal. It is important to review the preoperative computed tomography (CT) images and note the location of the rib head in relation to the disc space, pedicles above and below, and the central canal.

### 3:42 Removal of Pedicle and Partial Corpectomy.

Following removal of the rib head, attention is directed toward removing the pedicle below the disc space and achieving safe access to the thecal sac. An angled minimally invasive drill handle with a 4.5-mm course diamond burr is used. Once the dura is exposed, the anterior edge of the thecal sac is palpated to determine the safe zone to begin the corpectomy. The ultrasonic bone aspirator is again used to make a posterior cut just in front of the dura, as well as a more anterior cut. Fluoroscopy images are utilized to determine depth of the cut, which generally does not need to go past the contralateral pedicle. The 2 cranial-caudal cuts are connected superiorly and inferiorly, and that bone is removed using a pituitary rongeur. It is important to pull straight up without a twisting motion as to not push the herniated disc fragment posteriorly into the thecal sac.

The drill is then brought in to further expand the corpectomy, which includes the vertebral body above and the vertebral body below the disc space of interest. The goal will be to push the disc herniation anteriorly into this corpectomy defect and away from the dura. The posterior cortex of the vertebral body is thinned with a drill until reaching the posterior longitudinal ligament (PLL). The PLL is then dissected from the dura and cut across at the cranial and caudal portions of the corpectomy.

### 5:03 Discectomy.

At this point, attention is finally directed to the disc herniation itself, with the goal of finding a plane between the disc and the dura, and gently pushing the disc fragment anteriorly into the corpectomy defect. Once this fragment is in the corpectomy defect, it could be removed with pituitary rongeurs by pulling straight up and away from the thecal sac. Additional discectomy can be performed more anteriorly to the corpectomy site, and morselized rib autograft can be placed into the discectomy defect to provide in situ fusion. In case of a cerebrospinal fluid leak, a commercially available minimally invasive dural repair kit can be used to attempt primary repair. If primary repair is not feasible, secondary repair is done with a combination of dural sealant glue, followed by Gelfoam, followed by a lateral fixation plate with 2 screws. The plate is placed over the corpectomy defect to buttress the aforementioned repair materials in place and to prevent them from migrating into the retropleural space or chest cavity. If these measures are unsuccessful, a lumbar drain is an additional option.

### 6:05 Closure.

At this point attention is directed toward closure of the various muscular layers with 0 Vicryl sutures. An intercostal nerve block is administered at the level of the rib that was removed. A medium drain is left in the surgical field and tunneled out a separate site. The muscular layers are closed around a red rubber catheter, which is used to expel air and blood via Valsalva maneuvers. The red rubber catheter is then removed, and a previously placed purse string is tied at the site of where the catheter was.

### 6:35 Postoperative Course and Imaging.

Postoperative imaging demonstrates expected changes including removal of the rib head pedicle and vertebral bodies above and below. Initially, all of these cases were performed with pedicle screw fixation. However, over the last 50 cases we have reserved fixation only at the thoracolumbar junction, where there is increased mobility. In the thoracic spine, we do not perform pedicle screws and rely on fusion across the disc space, which is filled with autograft, as well as the intact posterior tension band, anterior longitudinal ligament, the contralateral annulus and pedicle, sternum, and rib cage. A chest x-ray is performed in the postoperative area to ensure there is no pneumothorax, and an additional chest x-ray is performed the next morning.^5^ Patients generally spend 1 night in the intensive care unit and are discharged by postoperative day 2.

Postoperative imaging demonstrates resolution of the prior disc herniation and stenosis, the expected partial corpectomy changes, and a widely patent canal.

Thank you for your attention.

## Acknowledgments

We thank the staff of Neuroscience Publications at Barrow Neurological Institute for assistance with manuscript and video preparation.

## Disclosures

Dr. Uribe serves as a consultant for Mainstay Medical, Viseon, Misonix, SI Bone, and NuVasive; and receives royalties from SI Bone as well as royalties, stock/stock options, and research support from NuVasive. Dr. Snyder serves as a consultant for Medtronic, Globus, and NuVasive; and has research support from Biogen.

## Author Contributions

Primary surgeon: Uribe. Assistant surgeon: Katsevman, Morgan. Editing and drafting the video and abstract: Uribe, Katsevman, Paisan, Snyder. Critically revising the work: Uribe, Katsevman, Paisan, Snyder. Reviewed submitted version of the work: Uribe, Katsevman, Paisan, Snyder. Approved the final version of the work on behalf of all authors: Uribe. Supervision: Uribe, Snyder.

## Supplemental Information

### Previous Presentations

Portions of this work were previously presented at the AANS/CNS Joint Section on Disorders of the Spine and Peripheral Nerves, Las Vegas, Nevada, February 23–26, 2022.

## References

[1] Yen CP Uribe JS Mini-open lateral retropleural approach for symptomatic thoracic disk herniations Clin Spine Surg 2018 31 1 14 21 28857966 10.1097/BSD.0000000000000580

[2] Wewel JT Uribe JS Retropleural thoracic approach Neurosurg Clin N Am 2020 31 1 43 48 31739928 10.1016/j.nec.2019.08.005

[3] Xu DS Walker CT Farber SH Surgical anatomy of minimally invasive lateral approaches to the thoracolumbar junction J Neurosurg Spine 2022 36 6 937 944 10.3171/2021.10.SPINE21793 34972082

[4] Christiansen PA Huang S Smith JS Shaffrey ME Uribe JS Yen CP Mini-open lateral retropleural/retroperitoneal approaches for thoracic and thoracolumbar junction anterior column pathologies Neurosurg Focus 2020 49 3 E13 10.3171/2020.6.FOCUS20360 32871570

[5] Uribe JS Smith WD Pimenta L Minimally invasive lateral approach for symptomatic thoracic disc herniation: initial multicenter clinical experience J Neurosurg Spine 2012 16 3 264 279 22176427 10.3171/2011.10.SPINE11291

